# Nomogram for prediction of the international study Group of Liver Surgery (ISGLS) grade B/C Posthepatectomy liver failure in HBV-related hepatocellular carcinoma patients: an external validation and prospective application study

**DOI:** 10.1186/s12885-020-07480-2

**Published:** 2020-10-28

**Authors:** Jia-zhou Ye, Rong-yun Mai, Wei-xing Guo, Yan-yan Wang, Liang Ma, Bang-de Xiang, Shu-qun Cheng, Le-qun Li

**Affiliations:** 1 Department of Hepatobiliary Surgery, Guangxi Medical University Cancer Hospital, Nanning, China; 2 Department of Hepatobiliary Surgery, Affiliated Tumour Hospital of Guangxi Medical University, Nanning, 530021 China; 3 Department of Hepatic Suegery VI, Eastern Hepatobiliary Surgery Hospital, Second Military Medical University, 225 Changhai Road, Shanghai, 200438 China; 4 Hepatopancreatobiliary Surgery Department I, Key Laboratory of Carcinogenesis and Translational Research, Ministry of Education, Peking University School of Oncology, Beijing Cancer Hospital and Institute, Beijing, China; 5 Department of Surgery, Prince of Wales Hospital, Chinese University of Hong Kong, Hong Kong, China; 6 National Research Cooperative Group for Diagnosis and Treatment of Hepatocellular Carcinoma with Tumour Thrombus, Shanghai, China

**Keywords:** Hepatocellular carcinoma, Hepatitis B virals, Posthepatectomy liver failure, Nomogram

## Abstract

**Background:**

To develop a nomogram for predicting the International Study Group of Liver Surgery (ISGLS) grade B/C posthepatectomy liver failure (PHLF) in hepatitis B virus (HBV)-related hepatocellular carcinoma (HCC) patients.

**Methods:**

Patients initially treated with hepatectomy were included. Univariate regression analysis and stochastic forest algorithm were applied to extract the core indicators and reduce redundancy bias. The nomogram was then constructed by using multivariate logistic regression, and validated in internal and external cohorts, and a prospective clinical application.

**Results:**

There were 900, 300 and 387 participants in training, internal and external validation cohorts, with the morbidity of grade B/C PHLF were 13.5, 11.0 and 20.2%, respectively. The nomogram was generated by integrating preoperative total bilirubin, platelet count, prealbumin, aspartate aminotransferase, prothrombin time and standard future liver remnant volume, then achieved good prediction performance in training (AUC = 0.868, 95%CI = 0.836–0.900), internal validation (AUC = 0.868, 95%CI = 0.811–0.926) and external validation cohorts (AUC = 0.820, 95%CI = 0.756–0.861), with well-fitted calibration curves. Negative predictive values were significantly higher than positive predictive values in training cohort (97.6% vs. 33.0%), internal validation cohort (97.4% vs. 25.9%) and external validation cohort (94.3% vs. 41.1%), respectively. Patients who had a nomogram score < 169 or ≧169 were considered to have low or high risk of grade B/C PHLF. Prospective application of the nomogram accurately predicted grade B/C PHLF in clinical practise.

**Conclusions:**

The nomogram has a good performance in predicting ISGLS grade B/C PHLF in HBV-related HCC patients and determining appropriate candidates for hepatectomy.

## Background

Hepatocellular carcinoma (HCC) is the sixth most common malignancy and the fourth leading cause of cancer-related death worldwide [1]. Hepatectomy is the most effective treatment for early-stage HCC patients, [2] and selective intermediate-stage and advanced-stage HCC patients with resectable tumors and moderate liver function [3]. Advances surgical techniques and management have greatly improved the safety and postoperative outcomes over the past few decades [4]; however, the International Study Group of Liver Surgery (ISGLS) grade B/C posthepatectomy liver failure (PHLF) remains a serious complication, which is a predominant cause of postoperative mortality [5, 6].

Incidence of PHLF as reported in literature widely ranges from 1.2–32% attributing to diverse etiological and pathogenic liver characteristics and surgical procedures [6, 7]. Independent risk factors of PHLF can be grouped into three categories [5, 8]: 1) Patient-related factors including age, sex, comorbidities such as malnutrition, diabetes mellitus, cardiopulmonary, renal or cerebral dysfunction; 2) liver disease-related factors including hepatitis B/C, steatosis, cholangitis, alcoholic liver disease and cirrhosis; 3) surgery-related factors including future liver remnant volume (FLRV), excessive intraoperative blood loss, prolonged operation time, and ischemia-reperfusion injury resulting from Pringle’s manoeuver manipulation. In particular, as a major cause to promote decompensate liver cirrhosis and dysfunction, chronic hepatitis B is highly prevalent and associated with 70–90% of HCC cases in the Asia-Pacific region [9].

Accurate prediction of PHLF is of primary concern for determining the feasibility of hepatectomy for HCC [5, 8]. Child-Pugh grade, [10] model for end-stage liver disease (MELD), [11] albumin–bilirubin (ALBI), [12, 13] platelet-albumin-bilirubin (PALBI) and aspartate aminotransferase to platelet ratio index (APRI) [14] are commonly conventional scores used for evaluating PHLF, nevertheless their predictive performance remains controversial due to inherent limitations. Child-pugh grade is the most widely used for evaluating compensate liver function and has been incorporated into surgical treatment algorithms [11]. However, subjective and unquantifiable variables usually complicate Child-pugh grade: serum bilirubin level of 55 μmol/L has the same influence on Child-pugh grade as 550 μmol/L due to arbitrary thresholds for continuous variables; there is no clear guideline for distinguishing mild or moderate ascites, and the influence of diuretic therapy on grading ascites remains unclear; sedatives therapy frequently mislead encephalopathy [15]. MELD was developed to evaluate acute liver failure mortality risk and rank candidates for transplantation, [16, 17] which was also good at determining increased PHLF morbidity and mortality risk when a MELD score > 8 on postoperative day 5 [5]. However, MELD has a poor performance at preoperative predicting PHLF [5, 6, 11]. ALBI statistical eliminates subjective observation and assesses liver function and overall survival compared favorably with Child-pugh grade in four geographical and etiological HCC patient groups [18]. A preoperative ALBI score predicting PHLF was more accurately than Child-pugh grade, MELD and indocyanine green retention at 15 min (ICG-15) [12, 13]. However, when patients with hyperbilirubinemia were divide into the ALBI grade 3, patients with obstructive jaundice may have better liver function and prognosis than patients with jaundice caused by decompensate liver dysfunction, which significantly misleading the grading of ALBI [19, 20]. Blood platelet (PLT) counts as a surrogate marker of portal hypertension was added to ALBI to develop the PALBI, which predicting survival in HCC patients across treatment modalities including hepatectomy was better than ALBI and MELD. Nonetheless, further research is necessary as few studies have been done evaluating use of PALBI for predicting PHLF. APRI is noninvasive and reliable for evaluating liver fibrosis and cirrhosis, [21, 22] meanwhile a high preoperative APRI score have a high risk of PHLF in HCC patients [23]. However, APRI only includes two quantitative variables and has no ceiling effect. In general, these conventional scores were primarily designed for assessing liver function or other purposes rather than predicting PHLF. Moreover, when they were used for predicting PHLF none of these scores comprehensively considers patient-related, liver-related, and surgical-related risk factors.

As an evidence-based model, nomogram has been proposed as an alternative tool for therapy risk individualized estimation in clinical application [24, 25]. This study aimed to establish a nomogram to predict grade B/C PHLF risk for HBV-HCC patients.

## Methods

### Patient population

This study was conducted retrospectively in HBV-related HCC patients who were initially treated with hepatectomy. The training and internal cohorts consisted of patients treated at the Guangxi Medical University Cancer Hospital (GXMUCH) between October 11th, 2013 and December 21st, 2017. The external cohort consisted of patients treated at the Eastern Hepatobiliary Surgery Hospital (EHBH) between September 14th, 2009 and January 22th, 2018. In addition, patients would receive hepatectomy as the initial treatment, were prospectively recruited from GXMUCH between December 22th, 2017 and June 21st, 2018 for evaluation of the nomogram in clinical application. This study was approved by the Institutional Ethics Committees of the two hospitals.

The inclusion criteria were as follows: (1) aged 18–75 years, (2) positive for hepatitis B surface antigen, (3) preoperative Eastern Cooperative Oncology Group (ECOG) performance score 0–2, (4) preoperative Child-pugh A/B liver function, and (5) histological diagnosis of HCC. Patients who had other simultaneous malignancies, hepatitis C, tumor rapture, extrahepatic metastasis, cardiopulmonary, renal or cerebral dysfunction, and underwent preoperative anticancer treatments were excluded.

### Definitions

ISGLS PHLF was diagnosed with an increased serum international normalized ratio (INR) and concomitant hyperbilirubinaemia after postoperative day 5 [5]. The severity of PHLF was graded as: grade A PHLF required no specific treatment; grade B PHLF required essential non-invasive treatment, such as fresh frozen plasma, administration of albumin and daily diuretics; grade C PHLF required invasive procedures including mechanical ventilation, hemodialysis or extracorporeal liver support. In this study, grade B and C PHLF were considered as severe PHLF [26, 27]. The standardized future liver remnant (sFLR) = FLR/estimated total liver volume (eTLV) was used to represent the percentage of postoperative residual liver. The equation calculating eTLV (cm^3^) = 706.2 × body surface area (BSA)(m^2^) + 2.4; BSA(m^2^) = 0.00607 × height (cm) + 0.0127 × weight (kg)-0.0698 for men and BSA(m^2^) = 0.00586 × height (cm) + 0.0126 × weight (kg)-0.0461 for women [28].

### Preoperative examination and surgical procedure

Preoperative general characteristics, laboratory biochemistry data (including liver and renal function tests, hepatitis immunology and serum α-fetoprotein level), radiological data (including abdominal contrast-enhanced CT or MRI scan, and chest radiograph), surgical data (including operation time, intraoperative blood loss, intraoperative transfusion, sFLR) were routinely collected. sFLR was determined by three-dimensional technology using software DEMedical (version3.1, DE Sci&Tech co., Ltd., Shenzhen, China). The details of the calculation of future liver remnant have been described in previous study [27]. Surgical procedures have been described in a previous report [3]. Pringle’s manoeuver was applied to occlude the liver blood. Electrosurgical instruments or clamp-crushing method was performed to carry out liver parenchymal transection. Histopathological examination was routinely conducted by three pathologists on all surgical specimens. The main outcome observed was the PHLF morbidity and mortality.

### Study design and statistical analyses

**Fig. 1:**
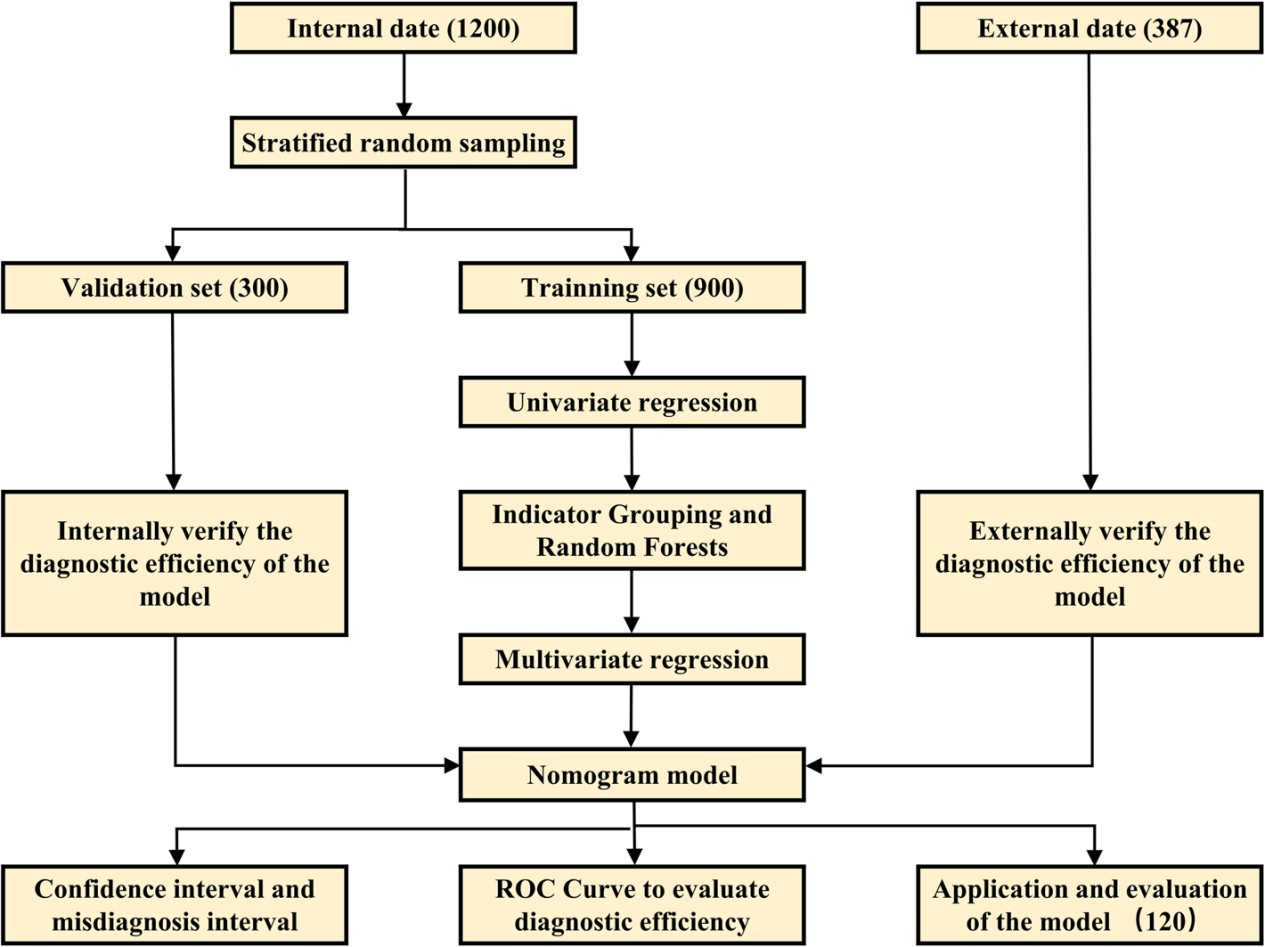
Flow chart of the study design

Flow chart of the study design is shown in Fig. 1. A stratified random grouping method was performed to randomly assign and divide patients into training cohort and internal validation cohort at a ratio of 3:1. In training cohort, logistic univariate analysis was used to identify independent risk indicators of grade B/C PHLF and correlation analysis was performed to eliminate data redundancy and excessive false positives. When correlation analysis indicated non-independence from univariate analysis, indicators were classified according to clinical significance in seven groups with different meanings (liver synthesis ability, metabolism ability, HBV activity status, liver inflammation, compensate cirrhotic liver function, coagulation function, and surgery-related factors). Stochastic According to the forest algorithm, indexes with the highest weight (at least> 20) in each category were extracted and incorporated into the subsequent logistic multivariate regression model. A nomogram was formulated using the RMS package in R version 3.3.2. The predictive performance of the nomogram was measured using receiver operating characteristic (ROC) curve and compared with conventional scores. Calibration plots methods evaluated the goodness of fit for the nomogram. Yoden index of the ROC curve from training cohort was calculated to set the diagnostic threshold. The diagnostic errors were displayed by correcting the curve. Area under the curve (AUC) represents the misdiagnosis threshold. Correspondingly, the confidence interval of diagnosis is expressed in abscissa of area beyond the 95% misdiagnosis threshold. For efficacy evaluation of the nomogram in prospective clinical application, total points of predictions were calculated for each patient, meanwhile statistical indicators including precision, recall, accuracy and F1 balance were calculated to evaluate the diagnostic ability.

Data analysis was performed using SPSS (Version 23.0, IBM, New York, USA) and R software (Version 3.2.2, Institute for Statistics and Mathematics, Vienna, VIC, Austria). Normally distributed continuous data are expressed as mean (s.d.) and compared using an unpaired. Two-tailed t-test Values with a non-normal distribution are expressed as median (IQR 25–75) and were compared using Mann-Whitney *U* test. Categorical data are shown as frequency and proportion and were compared using the χ^2^ test.

## Results

### Clinicopathologic characteristics

During the study period, 1200 HBV-related HCC patients from GXMUCH met the inclusion criteria were included and randomly assigned to a training cohort (*n* = 900) and an internal validation cohort (*n* = 300) at a ratio of 3:1. Besides, 387 HBV-related HCC patients from EHBH met the inclusion criteria were included in an external validation cohort (Fig. 1). Baseline clinicopathologic characteristics are listed in Table 1.

**Table 1:** Demographics and clinicopathologic characteristics of study participants

Variables	Training Cohort (*n* = 900)	Internal validation Cohort (*n* = 300)	External validation Cohort (*n* = 387)	*P* value
Age (years)	49 (42, 58)	49 (41, 55)	52 (45, 59)	<0.001
Sex, Male / Female	782 (86.9) / 118 (13.1)	261 (87.0) / 39 (13.0)	344 (88.9) / 43 (1.11)	<0.001
Height (cm)	166 (161, 170)	165 (160, 170)	165 (161, 170)	0.936
Weight (kg)	61 (55, 68)	60 (54, 67)	60 (54,69)	0.540
BMI (kg/m^2^)	22.3 (20.0, 24.4)	21.8 (20.0, 24.5)	22.1 (20.1, 24.5)	0.540
Hypertension, Yes / No	67 (7.4%) / 833 (92.6%)	19 (6.3%) / 281 (93.7%)	54 (14.0%) / 333 (86.0%)	<0.001
Diabetes, Yes / No	83 (9.2%) / 817 (90.8%)	21 (7.0%) / 279 (93.0%)	29 (7.5%) / 358 (92.5%)	0.373
HBsAg, Positive / Negative	259 (28.8%) / 641 (71.2%)	81 (27.0%) / 219 (73.0%)	90 (23.3%) / 297 (96.7%)	0.124
HBV-DNA, ≥2000 / < 2000 (IU/ml)	555 (61.7%) / 345 (38.4%)	179 (59.7%) / 121 (40.3%)	175 (45.2%) / 212 (54.8%)	<0.001
PLT (× 10^9^/L)	199.9 (155.0, 260.0)	207.1 (160.0, 261.7)	149.0 (100.0, 193.0)	<0.001
T-Bil (μmol/L)	12.9 (9.5, 17.2)	13.0 (9.7, 17.8)	14.2 (10.7, 18.9)	<0.001
PA (mg/L)	178.0 (135.0, 224.0)	173.0 (130.0, 217.0)	202.0 (150.0, 244.0)	<0.001
ALB (g/L)	39.6 ± 4.6	39.3 ± 4.8	41.5 ± 4.4	<0.001
ALT (U/L)	36.0 (24.0, 52.5)	34.0 (25.0, 49.0)	34.0 (24.0, 54.7)	0.788
AST (U/L)	40.0 (30.0, 58.0)	38.0 (29.0, 55.0)	34.1 (26.0, 54.0)	<0.001
CR (μmol/L)	77.0 (68.0, 88.0)	79.0 (68.0, 89.0)	69.0 (61.0, 78.0)	<0.001
BUN (mmol/L)	4.9 (4.1, 5.9)	5.0 (4.2, 5.9)	5.4 (4.5, 6.4)	<0.001
PT (s)	12.8 (12.1, 18.6)	13.0 (12.2, 18.0)	12.0 (11.5, 18.8)	<0.001
INR	1.05 (0.98, 1.13)	1.06 (1.00, 1.16)	1.01 (0.96, 1.07)	<0.001
AFP ≥400 / < 400 (ng/mL)	388 (43.1%) / 512 (56.9%)	142 (47.3%) / 158 (52.7%)	130 (33.6%) / 257 (66.4%)	<0.001
CSPH, Yes / NO	75 (8.3) / 825 (91.7)	31 (10.3) / 269 (89.7)	95 (24.5) / 292 (75.5)	<0.001
Child-Pugh grade A / B (7 score)	858 (95.3%) / 42 (4.7%)	284 (94.7%) / 16 (5.3%)	373 (96.4%) / 14 (3.6%)	0.541
MELD score	4 (2, 7)	5 (3, 7)	4 (2, 5)	<0.001
ALBI score	−2.63 ± 0.41	−2.61 ± 0.45	−2.76 ± 0.40	<0.001
PALBI score	−2.47 (−2.67, −2.25)	−2.43 (−2.67, −2.17)	−2.64 (−2.80, − 2.41)	<0.001
APRI score	0.52 (0.35, 0.84)	0.48 (0.34, 0.85)	0.64 (0.38, 1.19)	<0.001
Tumour size (cm)	6.1 (4.0, 17.5)	6.8 (4.0, 16.5)	5.7 (4, 18)	0.387
Tumour number, Multiple/ Single	239 (26.6%) / 661 (73.4%)	73 (24.3%) / 227 (75.7%)	101 (25.9%) / 286 (74.1%)	<0.001
Portal invasion, Yes / No	103 (11.4%) / 761 (84.6%)	29 (9.7%) / 271 (90.3%)	30 (7.7%) / 357 (92.3%)	0.393
BCLC stage				0.001
0	31 (3.4%)	12 (4.0%)	29 (7.5%)	
A	560 (62.2%)	197 (65.7%)	242 (62.5%)	
B	206 (22.9%)	62 (20.6%)	82 (21.3%)	
C	103 (11.5%)	29 (9.7%)	34 (8.7%)	
Operation time (min)	193 (160, 240)	200 (165, 250)	190 (160, 240)	0.172
Blood loss, ≥400 / < 400 (mL)	346 (38.4%) / 554 (61.6%)	114 (38.0%) / 186 (62.0%)	136 (35.1%) / 251 (64.9%)	<0.001
Blood transfusion, Yes / No	59 (6.6%) / 841 (93.4%)	21 (7.0%) / 279 (93.0%)	42 (10.9%) / 345 (89.1%)	0.026
eTLV (mL)	1212.2 ± 109.6	1208.6 ± 107.5	1212.0 ± 113.0	0.874
FLR (mL)	939.6 (791.2, 1042.5)	919.8 (780.8, 1028.8)	948.2 (840.6, 1045.9)	0.053
sFLR (%)	69.0 (34.0, 85.0)	67.9 (36.0, 84.2)	69.6 (37.0, 86.0)	0.013
Hepatic vascular occlusion				0.903
No	243 (27.0%)	80 (26.7%)	111 (28.7%)	
HVC	283 (31.4%)	89 (29.7%)	114 (29.5%)	
THVE	374 (41.6%)	131 (43.7%)	162 (41.8%)	
Cirrhosis, Yes / No	389 (43.2%) / 511 (56.8%)	149 (49.7%) / 151 (50.3%)	190 (49.1%) / 197 (51.9%)	0.052
PHLF	214 (23.8%) / 686 (76.2%)	63 (21.0%) / 237 (79.0%)	145 (37.5) / 242 (62.5%)	<0.001
Grade A	93 (10.3%)	30 (10.0%)	67 (17.3%)	<0.001
Grade B	114 (12.7%)	32 (10.7%)	70 (18.1%)	
Grade C	7 (0.8%)	1 (0.3%)	8 (2.1%)	
Grade B/C	121 (13.5%)/779 (86.5%)	33 (11.0%) / 267 (89.0%)	78 (20.2%) / 309 (79.8%)	0.001

Note: Data are mean ± SD or median (IQR 25–75) unless otherwise indicated

Abbreviations: *BMI* Body mass index, *HbsAg* Hepatitis B surface antigen, *HBV-DNA* Hepatitis B virus DNA, *PLT* Platelet, *T-Bil* Total bilirubin, *PA* Prealbumin, *ALB* Albumin, *ALT* Alanine aminotransferase, *AST* Aspartate aminotransferase, *CR* Creatinine, *BUN* Blood urine nitrogen, *PT* Prothrombin time, *INR* International normalized ratio, *MELD* Model for end-stage liver disease, *ALBI* Albumin–bilirubin, *PALBI* Platelet-albumin-bilirubin, *APRI* Aspartate aminotransferase to platelet ratio index, *AFP* α-Fetoprotein, *CSPH* Clinically signifcant portal hypertension, *BCLC* Barcelona Clinic Liver Cancer, *eTLV* Estimated total liver volume, *FLR* Future liver remnant, *sFLR* Standard future Liver remnant, *HVC* Hemilhepatic vascular control, *THVE* Total hepatic vascular exclusion, *PHLF* Posthepatectomy liver failure

The incidences of grade B/C PHLF were 13.5, 11.0 and 20.2% in the training cohort, internal cohort and external cohort, respectively (Table 1). The postoperative mortality rate of entire participants at GXMUCH was 1.75% (21 patients): 13 patients died as a result of grade C PHLF following multiple organ failure, 8 patients died due to sepsis or severe pneumonia. The postoperative mortality rate of participants from EHBH was 0%. In participants for prospective clinical application of the nomogram, grade B PHLF occurred in 14 patients (11.7%) and grade C PHLF occurred in 4 patients (3.3%).

### Independent risk indicator of ISGLS grade B/C PHLF

**Fig. 2:**
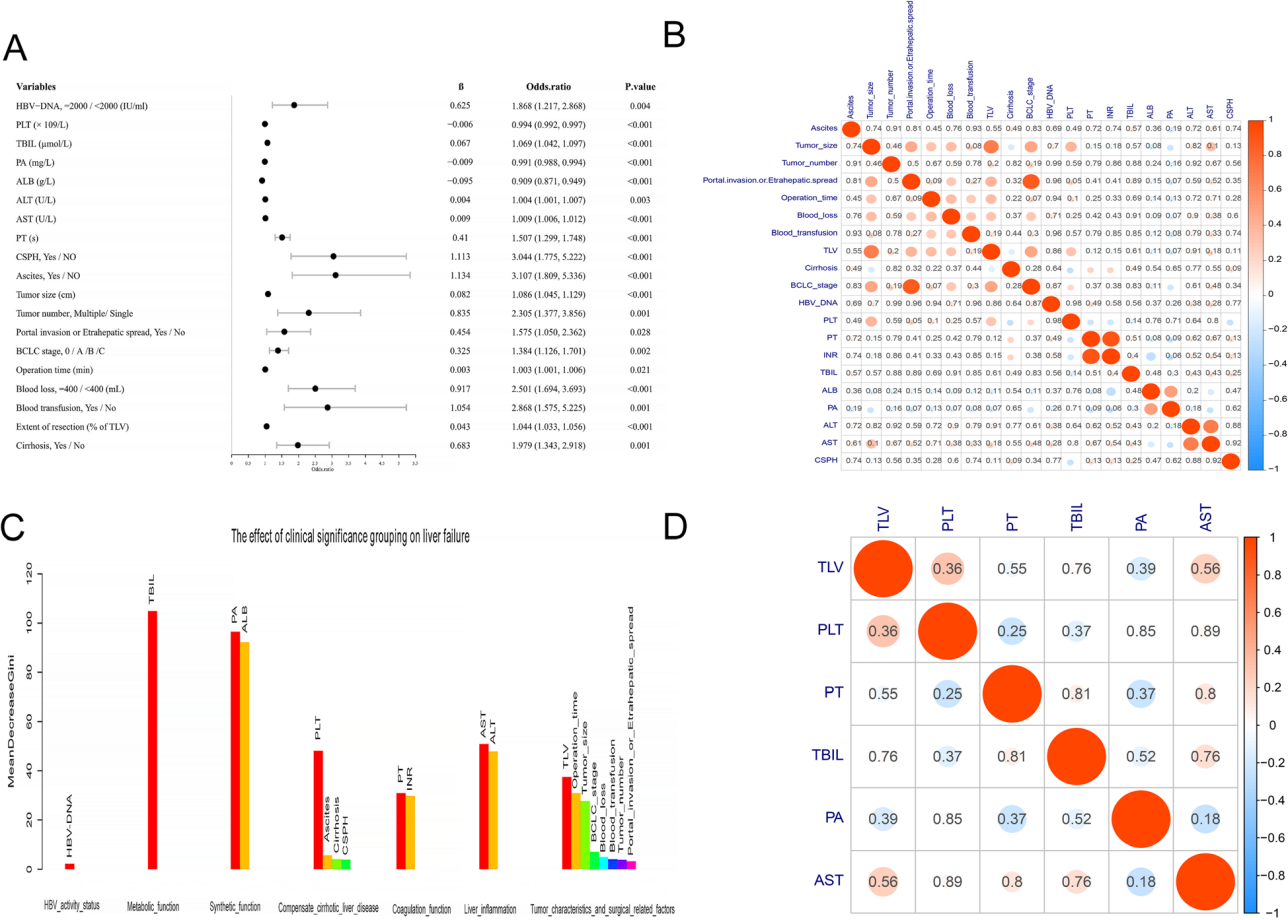
Univariable logistic regression analyses to identify predictors of Grade B/C PHLF in patients with HBV-related HCC in the training cohort. Forest maps show the risk ratios of indicators. **b** Correlation analysis among indicators significantly related with grade B/C PHLF by logistic univariate analysis. Colors from red to blue indicate a correlation from positive to negative. The values represent the significant *P* values of the correlations, indicating the parts of correlations are significant. **c** The importance of the Stochastic Forest algorithm based on grouping indexes. Logistic univariate significant indicators were divided into seven groups according to clinical significance and a random forest model was constructed for each group of indicators to predict grade B/C PHLF risk. The bars represent the importance of each indicator; the red bars represent the most important indicators of each group. **d** There is no correlation among the indicators after redundancy removal by grouping stochastic forest algorithm. Colors from red to blue indicate a correlation from positive to negative. The values inside the circle represent the significant *P* values of the correlations, indicating the correlations among all indicators are not significant

In training cohort, independent risk indicators of grade B/C PHLF was identified by logistic univariate analysis (Fig. 2a) and data redundancy and excessive false positives were eliminated by correlation analysis (Fig. 2b). According to the forest algorithm, serum HBV-DNA loads with weights of 2.19 were excluded (Fig. 2c). Multivariate analysis for a stepwise removal of variables was then done, and the results reported as odds ratios with 95%CI, total bilirubin (T-Bil), platelet (PLT) count, prealbumin (PA), aspartate aminotransferase (AST), prothrombin time (PT), and sFLR were identified independently influenced on grade B/C PHLF (Table 2). Then, the second correlation test revealed that not significant independence among these six independent indicators, which are able to be incorporated into the nomogram (Fig. 2d).

**Table 2:** Multivariable logistic regression analyses of grade B/C PHLF in the training cohort

Variables	β	Odds ratio	*P* value
T-Bil (μmol/L)	0.040	1.041 (1.015, 1.068)	0.002
PLT (× 10^9^/L)	−0.010	0.990 (0.987, 0.993)	<0.001
PA (mg/L)	−0.005	0.995 (0.992, 0.999)	0.019
AST(U/L)	0.004	1.004 (1.000, 1.008)	0.035
PT (s)	0.318	1.375 (1.144, 1.652)	<0.001
sFLR (%)	−0.067	0.936 (0.926, 0.949)	<0.001

Abbreviations: *TBIL* Total bilirubin, *PLT* Platelet, *PA* Prealbumin, *AST* Aspartate aminotransferase, *PT* Prothrombin time, *sFLR* Standard future liver remnant

### Development and validation of a grade B/C PHLF-predicting nomogram

**Fig. 3:**
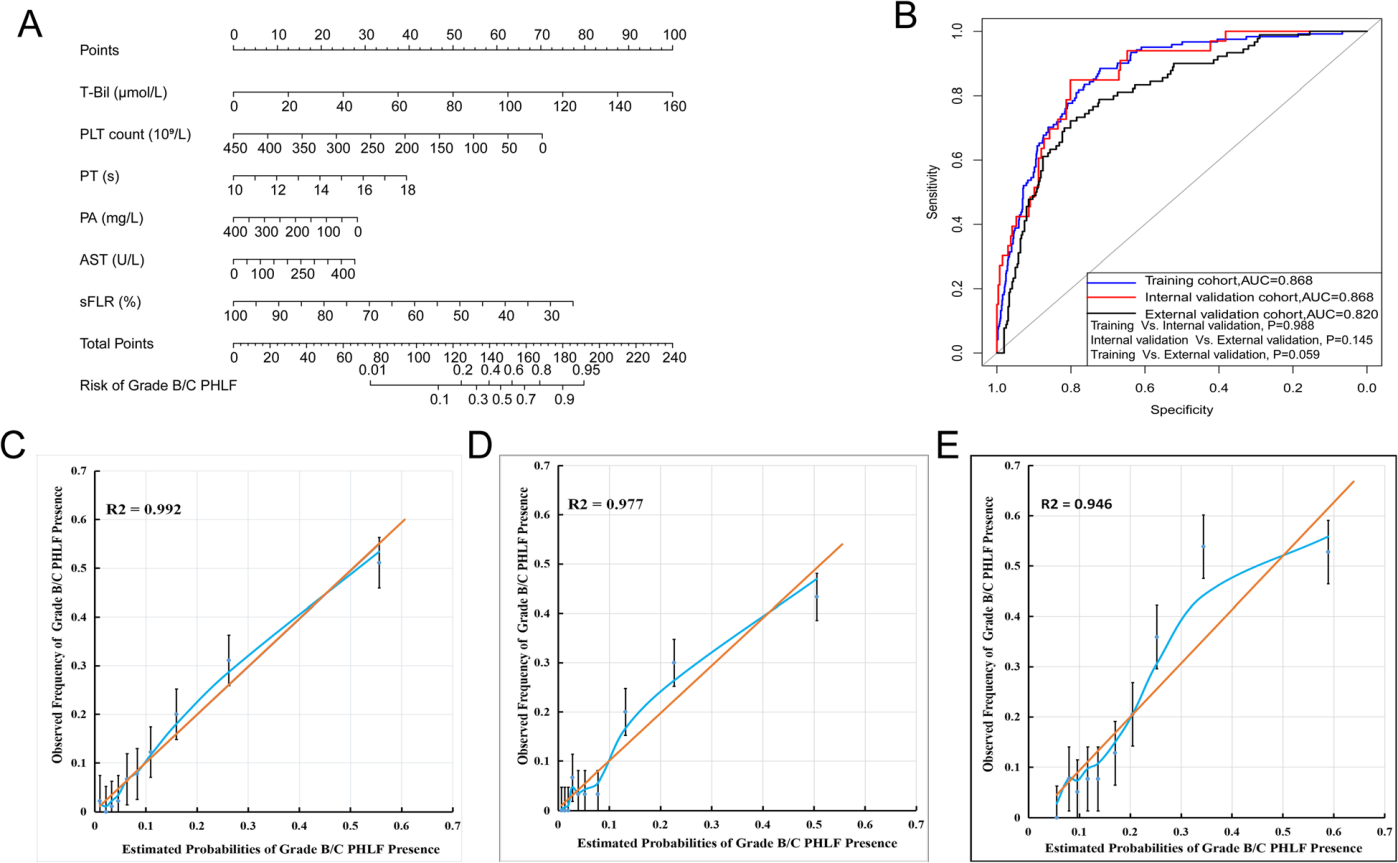
**a** Nomogram for predicting grade B/C PHLF in HBV-related HCC patients. To use the nomogram, find the position of each variable on the corresponding axis, draw a line to the points axis for the number of points, add the points from all of the variables, and draw a line from the total points axis to determine the grade B/C PHLF probabilities at the lower line of the nomogram. **b** Receiver operating characteristic (ROC) curves for the nomogram in predicting grade B/C PHLF. Calibration plots show the relationship between the predicted probabilities based on the nomogram and actual values: **c** Training cohort, **d** Internal validation cohort, **e** External validation cohort. Nomogram-predicted probability of grade B/C PHLF is plotted on the x-axis, and the actual probability is plotted on the y-axis

Based on the multivariate logistic analysis (Table 2), a nomogram integrating T-Bil, PLT, PA, AST, PT and sFLR was developed (Fig. 3a). ROC curve was applied to evaluate accuracy of the grade B/C PHLF prediction. The area under the ROC curve (AUC) of the nomogram was 0.868 (95%CI: 0.836–0.900) in training cohort, 0.868 (95%CI: 0.811–0.926) in internal validation cohort, and 0.820 (95%CI: 0.756–0.861) in external validation cohort (Fig. 3b). Differences among the three cohorts were not statistically significant. In training cohort, the optimal cutoff value of total points to predict grade B/C PHLF was determined to be 169, with a sensitivity, specificity, positive predictive value, and negative predictive value of 88.4, 72.1, 33.0, and 97.6%, respectively. Bootstrap validation results showed good performance, with a sensitivity, specificity, positive predictive value, and negative predictive value of 84.8, 70.0, 25.9, and 97.4% in internal validation; and 83.3, 69.9, 41.1, and 94.3% in external validation cohort, respectively (Table 3). Calibration curves assessing risk and analyzing consistency of results showed good agreement for probability of grade B/C PHLF between the actual observation and prediction in training cohort (R^2^ = 0.992), internal validation (R^2^ = 0.977) and external validation cohorts (R^2^ = 0.946) (Fig. 3c–e).

**Table 3:** Accuracy of the prediction score of the Nomogram for estimating the risk of grade B/C PHLF incidence

Variable	Value (95% CI)		
	Training cohort	Internal validation cohort	External validation cohort
Area under ROC curve	0.868 (0.836 to 0.900)	0.868 (0.811 to 0.926)	0.820 (0.756 to 0.861)
Cutoff score	169	169	169
Sensitivity, %	88.4 (81.0 to 93.3)	84.8 (67.3 to 94.3)	83.3 (72.8 to 90.5)
Specificity, %	72.1 (68.8 to 75.2)	70.0 (64.1 to 75.4)	69.9 (64.4 to 74.9)
Positive predictive value, %	33.0 (28.0 to 38.5)	25.9 (18.2 to 35.4)	41.1 (33.5 to 49.3)
Negative predictive value, %	97.6 (95.9 to 98.6)	97.4 (93.7 to 99.0)	94.3 (90.3 to 96.8)
Positive likelihood ratio	3.17 (2.79 to 3.62)	2.83 (2.24 to 3.58)	2.77 (2.27 to 3.37)
Negative likelihood ratio	0.16 (0.10 to 0.26)	0.22 (0.10 to 0.49)	0.24 (0.14 to 0.39)

**Fig. 4:**
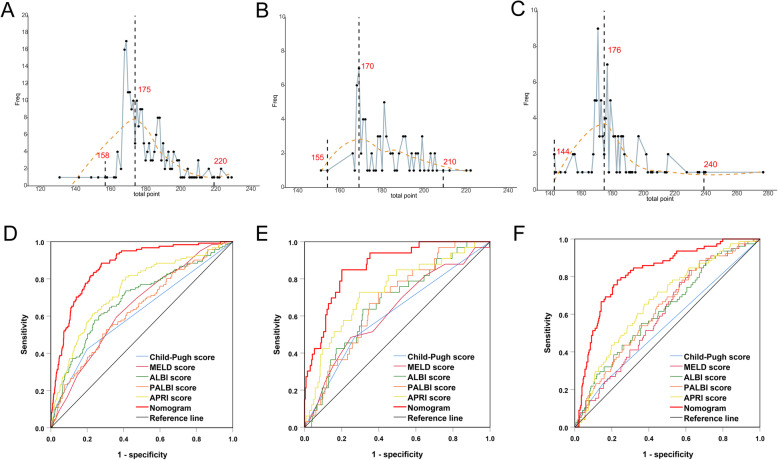
**a** Total points distribution of false positive events (blue polyline). The X-axis represents the total points used to predict the risk of grade B/C PHLF, the Y-axis represents the frequency of false positive events. The red dotted line represents the fitted line and presents a normal distribution. **a** Training cohort, the false positive events were concentrated around the maximum value 175 point, and close to the preset cutoff (169 points). **b** Internal validation cohort, the false positive events were concentrated around the maximum value 170 points. **c** External validation cohort, the false positive events were concentrated around the maximum value 176 points. **b** Comparison of predicative performance for predicting grade B/C PHLF between the nomogram and conventional scores: **a** Training cohort, **b** Internal validation cohort, **c** External validation cohort

Further, calculations of objectivity evaluation of diagnostic confidence interval revealed that total points of diagnostic errors with 95%CI were concentrated in 175 (ranged 158–220) in training cohort; concentrated in 170 (ranged 155–210) in internal validation cohort and concentrated in 176 (ranged 144–240) in external validation cohort (Fig. 4a-c), respectively. The sizes of confidence intervals among the three cohorts were very similar and the positions of the concentrated total points were close to the best cutoff value of 169. Confidence interval is considered to be prediction of low risk of grade B/C PHLF when total points fall below this range, while to be prediction of high risk of grade B/C PHLF when total points beyond this range. However, when total points fall within this range, the prediction results should be carefully considered.

### Comparison of predictive accuracy for grade B/C PHLF among the nomogram and conventional scores

When compared with conventional scores, the nomogram had greater discriminatory performance for predicting grade B/C PHLF than Child-pugh grade, MELD, ALBI, PALBI and APRI in training cohort, internal validation cohort and external validation cohort (Fig. 4d-f, Table 4 and Supplemental Table 1), which was not significantly influenced by inherent heterogeneity in different cohorts.

**Table 4:** Discriminatory performance of conventional scores and the nomogram for predicting grade B/C PHLF

	Training Cohort			Internal validation Cohort			External validation Cohort		
	AUC	95% CI	*P* value	AUC	95% CI	*P* value	AUC	95% CI	*P* value
Child-Pugh	0.616	0.558–0.674	<0.001	0.608	0.502–0.715	0.043	0.541	0.467–0.615	0.267
MELD	0.647	0.597–0.697	<0.001	0.627	0.523–0.731	0.017	0.601	0.535–0.667	0.006
ALBI	0.689	0.635–0.743	<0.001	0.667	0.574–0.760	0.002	0.620	0.551–0.689	0.001
PALBI	0.634	0.580–0.687	<0.001	0.667	0.590–0.762	<0.001	0.668	0.603–0.732	<0.001
APRI	0.741	0.692–0.789	<0.001	0.734	0.640–0.827	<0.001	0.626	0.560–0.692	0.001
Nomogram	0.868	0.836–0.900	<0.001	0.867	0.809–0.925	<0.001	0.820	0.756–0.861	<0.001

Abbreviations: *MELD* Model for end-stage liver disease, *ALBI* Albumin–bilirubin, *PALBI* Platelet-albumin-bilirubin, *APRI* Aspartate aminotransferase to platelet ratio index

### Prospective clinical application of the nomogram to predict grade B/C PHLF

In order to further evaluate the ability of predicting grade B/C PHLF in clinical application, the nomogram was applied to predict whether grade B/C PHLF occurred in 120 individual HBV-related HCC patients who would receive hepatectomy in GXCH. As a result, we accurately predicted that 85 patients would not have grade B/C PHLF, while 16 patients would; the remaining 19 cases were misjudged with total points were within 165–197 (Supplemental Table 1). All miscalculations have been re-evaluated and incorrect predicted total points (ranged 165–197) are fully contained within 158–220. The results of empirical evaluation of confidence interval help to improve the practicability of the nomogram and support the scientific of the study design. In addition, the predictive performance of the nomogram for judging non-occurrence of grade B/C PHLF is good in clinical practice, with a precision of 0.977, a recall of 0.833, an accuracy of 0.947, and a F1 balanced Score of 0.899.

## Discussion

In this study, after eliminating data redundancy and excessive false positives (Fig. 2), a nomogram was developed by integrating five essential preoperative serum laboratory biochemistries (T-Bil, PLT, PA, AST, PT) and sFLR from different categories with clinical significance comprehensively indicating compensate liver function and the percentage of postoperative remain liver (Table 2). Then a graphical and easy-to-use tool was applied for individualized predicting grade B/C PHLF in HBV-HCC patients (Fig. 3a). This nomogram displayed a good accuracy of prediction for grade B/C PHLF (Fig. 3b) and good agreement between probability and actual observation in training cohort, internal validation cohort and external validation cohort (Fig. 3c-e). Besides, this nomogram had greater predicative performance than conventional scores (Fig. 4a-c, Table 4). Further, calculations of predictive value (95% CIs) of the nomogram revealed that negative predictive values were significantly higher than positive predictive values in training cohort (97.6% vs. 33.0%), internal validation cohort (97.4% vs. 25.9%) and external validation cohort (94.3% vs. 41.1%) (Table 4), respectively, potentially demonstrated that negative predictive values could be precise for predicting grade B/C PHLF. Considering the PHLF can indeed be misdiagnosed, objective evaluation of the diagnostic ability of the nomogram and identification of confidence intervals were conducted. Results from feedback error diagnosis distribution curves revealed that actual distribution of total points for error prediction with 95%CI was concentrated in 175 (158–220), 170 (155–210) and 176 (144–240) in training cohort, internal validation and external validation cohorts, respectively, and were very close to the best cutoff value of 169 (Fig. 4a-c). Such that doctors have more flexibility when using the diagnostic results. Confidence interval is considered to be prediction of low risk of grade B/C PHLF when total points fall below this range and high risk of grade B/C PHLF when total points beyond this range. While total points fall within this range, the prediction results should be carefully considered and further confirmed through other evaluations such as ICG-15 retention or computer residual liver imaging volumetry. In this study, clinical use of the nomogram was evaluated in 120 HBV-related HCC patients who will receive hepatectomy. We found that preoperative application of this nomogram had good predictive performance for acutely judging grade B/C PHLF did not occured in 85 patients; and occurred in 16 patients; misjudged in 19 cases with total points were fall within 165–197, which were all fall within the 95%CI of diagnostic errors between 158 and 220 (Supplemental Table 1). The scoring range of misjudged cases was consistent with the objective evaluation, which supports the scientific nature of this study and guides further flexible application of this nomogram. In addition, this nomogram had a good predictive performance for judging non-occurrence of grade B/C PHLF in clinical practice, which was consistent with negative predictive values analysis.

A growing body of researches confirmed that major hepatectomy and insufficient sFLR was associated with high risk of PHLF [27, 29]. Our result is consistent with previous findings revealed that sFLR in this nomogram is of great importance for predicting grade B/C PHLF and adopted sFLR as an important predictive indicator of grade B/C PHLF in our model [30]. Recently, two radiomics-based nomograms based on portal-phase computed tomography or ultrasound were established to predict PHLF, [31, 32] however, they didn’t considered the influence of sFLR on PHLF and integrated this indicator. Moreover, considering mild-moderate liver dysfunction after liver surgery is very common, these nomograms remains to be further validated for predicting ISGLS grade B/C PHLF, particular in HBV-related HCC patients.

This study remains some limitations. First, all of the study participants were associated with HBV, while in most western countries and Japan, the majority of HCC cases are related to alcoholic liver disease or HCV. Therefore, further validation is required from other etiological populations. Second, the reliability of the nomogram remains to be further confirmed by conducting prospective and multicenter validation studies with expanding study participants. Moreover, advanced imaging scans and ICG-15 retention to estimate PHLF might be taken into further consideration to improve the diagnostic value.

## Conclusions

By comprehensively integrating five essential preoperative serum laboratory biochemistries (T-Bil, PLT, PA, AST, PT) and sFLR with different clinical significance comprehensively indicating the compensate liver function and the percentage of remain liver after hepatectomy, a novel nomogram was generated for individualized predicting ISGLS grade B/C PHLF in HBV-HCC patients. The results of internal and external validations demonstrated that this nomogram had good predicative performance. Prospective clinical application of this nomogram proposed an accurate judgment of non-occurrence of ISGLS grade B/C PHLF. It potentially provides an alternative tool for determining HBV-HCC patients with low risk of ISGLS grade B/C PHLF are appropriate candidates for hepatectomy.

## Supplementary information

**Additional file 1.** Supplement Table 1. Correlation analysis of 120 individual HBV-HCC patients’ grade B/C PHLF risk prediction data by the nomogram in prospective clinical application.

## Data Availability

All data within the article and supplementary files are available for publish. All the raw data is publicly available or can be requested from the first or correspondence authors.

## Abbreviations

HCC: Hepatocellular carcinoma
ISGLS: International Study Group of Liver Surgery
PHLF: Post-hepatectomy liver failure
sFLR: Standard future liver remnant
MELD: Model for end-stage liver disease
ALBI: Albumin–bilirubin
PALBI: Platelet-albumin-bilirubin
APRI: Aspartate aminotransferase to platelet ratio index
ECOG: Eastern Cooperative Oncology Group
