# Supplementary material for: Nomogram for prediction of the international study Group of Liver Surgery (ISGLS) grade B/C Posthepatectomy liver failure in HBV-related hepatocellular carcinoma patients: an external validation and prospective application study
Source: BMC Cancer. 2020 Oct 28;20:1036. doi: 10.1186/s12885-020-07480-2 (PMC7592579; doi:10.1186/s12885-020-07480-2)
Supplement: Supplementary file 1 — Additional file 1. Supplement Table 1. Correlation analysis of 120 individual HBV-HCC patients’ grade B/C PHLF risk prediction data by the nomogram in prospective clinical application. [file 12885_2020_7480_MOESM1_ESM.docx]

Supplement table 1. Correlation analysis of 120 individual HBV-HCC patients’ grade B/C PHLF risk prediction data by the nomogram in prospective clinical application

| Patients’ ID | Total Points | Prediction | Reality | Diagnosis |
| --- | --- | --- | --- | --- |
| 136950 | 162.8456604 | 0 | 0 | 1 |
| 120817 | 180.5198643 | 1 | 0 | 0 |
| 138877 | 170.1343351 | 1 | 1 | 1 |
| 147969 | 136.8018591 | 0 | 0 | 1 |
| 147988 | 189.5190713 | 1 | 1 | 1 |
| 148097 | 231.0624672 | 1 | 1 | 1 |
| 148219 | 158.9694733 | 0 | 0 | 1 |
| 148270 | 138.1818442 | 0 | 0 | 1 |
| 148315 | 167.3403649 | 0 | 0 | 1 |
| 148383 | 152.7362701 | 0 | 0 | 1 |
| 148408 | 186.5218316 | 1 | 1 | 1 |
| 148734 | 161.9212017 | 0 | 0 | 1 |
| 148869 | 137.4840548 | 0 | 0 | 1 |
| 148949 | 145.4586717 | 0 | 0 | 1 |
| 148981 | 191.6037517 | 1 | 1 | 1 |
| 148997 | 166.9851706 | 0 | 0 | 1 |
| 149023 | 137.3290853 | 0 | 0 | 1 |
| 149089 | 161.010228 | 0 | 0 | 1 |
| 149104 | 153.7135177 | 0 | 0 | 1 |
| 149106 | 175.7175549 | 1 | 0 | 0 |
| 149121 | 147.0124319 | 0 | 0 | 1 |
| 149142 | 215.183614 | 1 | 1 | 1 |
| 149154 | 141.1986725 | 0 | 0 | 1 |
| 149251 | 136.3089289 | 0 | 0 | 1 |
| 149361 | 141.1451368 | 0 | 0 | 1 |
| 149423 | 171.7664828 | 1 | 1 | 1 |
| 149496 | 197.1863885 | 1 | 0 | 0 |
| 149530 | 128.5170084 | 0 | 0 | 1 |
| 149543 | 181.0445881 | 1 | 0 | 0 |
| 149656 | 150.0076975 | 0 | 0 | 1 |
| 149675 | 150.0209265 | 0 | 0 | 1 |
| 149725 | 177.9925431 | 1 | 1 | 1 |
| 149758 | 167.9113378 | 0 | 1 | 0 |
| 150141 | 211.8217215 | 1 | 1 | 1 |
| 150156 | 145.9624029 | 0 | 0 | 1 |
| 150160 | 156.7340712 | 0 | 0 | 1 |
| 150186 | 128.5553841 | 0 | 0 | 1 |
| 150271 | 173.3829752 | 1 | 1 | 1 |
| 150290 | 125.4044404 | 0 | 0 | 1 |
| 150329 | 150.3319623 | 0 | 0 | 1 |
| 150425 | 179.0349873 | 1 | 0 | 0 |
| 150456 | 143.7896346 | 0 | 0 | 1 |
| 150474 | 124.193939 | 0 | 0 | 1 |
| 150491 | 147.2709325 | 0 | 0 | 1 |
| 150527 | 170.9259227 | 1 | 0 | 0 |
| 150536 | 179.020728 | 1 | 0 | 0 |
| 150641 | 165.038835 | 0 | 1 | 0 |
| 150718 | 180.9077234 | 1 | 0 | 0 |
| 150789 | 166.4107238 | 0 | 0 | 1 |
| 150864 | 184.0864155 | 1 | 1 | 1 |
| 151158 | 154.89701 | 0 | 0 | 1 |
| 151241 | 154.0712919 | 0 | 0 | 1 |
| 151289 | 158.5938109 | 0 | 0 | 1 |
| 151421 | 189.8284629 | 1 | 1 | 1 |
| 151432 | 182.2818152 | 1 | 0 | 0 |
| 151491 | 150.1173782 | 0 | 0 | 1 |
| 151502 | 172.8773139 | 1 | 0 | 0 |
| 151533 | 146.1153384 | 0 | 0 | 1 |
| 151617 | 148.802845 | 0 | 0 | 1 |
| 151672 | 118.6820377 | 0 | 0 | 1 |
| 151718 | 156.1779981 | 0 | 0 | 1 |
| 151728 | 164.4894508 | 0 | 0 | 1 |
| 151780 | 162.8834151 | 0 | 0 | 1 |
| 151856 | 148.2010256 | 0 | 0 | 1 |
| 151858 | 148.3344197 | 0 | 0 | 1 |
| 151928 | 138.4281195 | 0 | 0 | 1 |
| 151966 | 152.9300196 | 0 | 0 | 1 |
| 152024 | 162.4833039 | 0 | 0 | 1 |
| 152042 | 171.2579874 | 1 | 0 | 0 |
| 152101 | 176.7878524 | 1 | 0 | 0 |
| 152104 | 142.7057834 | 0 | 0 | 1 |
| 152130 | 167.5359883 | 0 | 0 | 1 |
| 152131 | 159.4824265 | 0 | 0 | 1 |
| 152168 | 229.9710244 | 1 | 1 | 1 |
| 152194 | 164.8435757 | 0 | 0 | 1 |
| 152246 | 169.3119622 | 1 | 0 | 0 |
| 152265 | 134.512716 | 0 | 0 | 1 |
| 152291 | 141.1866853 | 0 | 0 | 1 |
| 152308 | 172.0256208 | 1 | 0 | 0 |
| 152320 | 163.9702026 | 0 | 0 | 1 |
| 152388 | 153.2252557 | 0 | 0 | 1 |
| 152413 | 152.1069026 | 0 | 0 | 1 |
| 152610 | 529.919601 | 1 | 1 | 1 |
| 152673 | 186.109683 | 1 | 0 | 0 |
| 152675 | 139.2824296 | 0 | 0 | 1 |
| 152689 | 156.8508382 | 0 | 0 | 1 |
| 152699 | 128.618733 | 0 | 0 | 1 |
| 152714 | 163.8567322 | 0 | 0 | 1 |
| 152725 | 164.0566451 | 0 | 0 | 1 |
| 152732 | 165.8350372 | 0 | 0 | 1 |
| 152810 | 145.6715884 | 0 | 0 | 1 |
| 152856 | 157.8407939 | 0 | 0 | 1 |
| 152947 | 145.1680071 | 0 | 0 | 1 |
| 153019 | 179.8637287 | 1 | 0 | 0 |
| 153068 | 158.5084217 | 0 | 0 | 1 |
| 153096 | 140.052891 | 0 | 0 | 1 |
| 153164 | 147.3015465 | 0 | 0 | 1 |
| 153321 | 146.2470457 | 0 | 0 | 1 |
| 153336 | 162.0716303 | 0 | 0 | 1 |
| 153337 | 141.4816326 | 0 | 0 | 1 |
| 153372 | 201.6259352 | 1 | 1 | 1 |
| 153384 | 152.4298252 | 0 | 0 | 1 |
| 153448 | 146.1482906 | 0 | 0 | 1 |
| 153513 | 134.8741662 | 0 | 0 | 1 |
| 153592 | 161.9845339 | 0 | 0 | 1 |
| 153610 | 144.8153525 | 0 | 0 | 1 |
| 153624 | 125.326377 | 0 | 0 | 1 |
| 153646 | 155.9592851 | 0 | 0 | 1 |
| 153657 | 156.8780054 | 0 | 0 | 1 |
| 153703 | 144.4865361 | 0 | 0 | 1 |
| 153711 | 158.736471 | 0 | 0 | 1 |
| 153804 | 141.9551705 | 0 | 0 | 1 |
| 153826 | 108.8258882 | 0 | 0 | 1 |
| 153840 | 147.1239362 | 0 | 0 | 1 |
| 153926 | 141.2037503 | 0 | 0 | 1 |
| 153927 | 132.2175385 | 0 | 0 | 1 |
| 154051 | 161.0925616 | 0 | 0 | 1 |
| 154070 | 182.479762 | 1 | 1 | 1 |
| 154120 | 123.5895229 | 0 | 0 | 1 |
| 154169 | 175.3841908 | 1 | 0 | 0 |
